# Follicular Dendritic Cell Sarcoma Complicated With Preeclampsia and Fetal Growth Restriction in Pregnancy

**DOI:** 10.1155/carm/6362169

**Published:** 2025-11-30

**Authors:** Mehmet Can Keven, Güldal Esendağlı, Melike Savaş, Ece Aydoğdu, Ayşenur Sert, Banu Derim Yeğen, Özer Birge, Hasan Bostancı

**Affiliations:** ^1^Department of Gynecology Obstetrics, Division of Perinatology, Eskişehir City Hospital, Eskişehir, Turkey; ^2^Department of Medical Pathology, Faculty of Medicine, Gazi University, Ankara, Turkey; ^3^Department of Gynecology Obstetrics, Eskişehir City Hospital, Eskişehir, Turkey; ^4^Department of Gynecology Obstetrics, Division of Gynecologic Oncology, Eskişehir City Hospital, Eskişehir, Turkey; ^5^Department of General Surgery, Faculty of Medicine, Gazi University, Ankara, Turkey

**Keywords:** fetal growth restriction, follicular dendritic cell sarcoma, preeclampsia, pregnancy

## Abstract

Follicular dendritic cell sarcoma (FDCS) is a rare neoplasm of mesenchymal origin arising from B-cell follicles, typically presenting as a painless, slow-growing mass. According to the available literature, this is only the second reported case of FDCS during pregnancy and the first associated with fetal growth restriction and preeclampsia. A 30-year-old pregnant woman at 28 weeks of gestation was diagnosed with preeclampsia and fetal growth restriction, during which an incidental abdominal mass was identified on ultrasound. Excisional surgery was performed 27 days after an emergency cesarean delivery, and histopathological analysis confirmed FDCS arising in association with hyaline-vascular type Castleman disease. This case highlights the importance of multidisciplinary evaluation and awareness of rare abdominal neoplasms during pregnancy.

## 1. Introduction

Follicular dendritic cells (FDCs) are mesenchymal-derived cells located in B-cell follicles, where they capture, retain, and present antigens to surrounding B cells [1]. Primary malignant neoplasms with features of FDCs were first identified in 1986 [2]. FDC sarcoma (FDCS) is a rare neoplasm arising in both lymph nodes and extranodal sites, characterized by the morphological and immunophenotypic features of FDCs [1, 3, 4].

FDCS may occur sporadically or in association with hyaline-vascular type Castleman disease (CD), suggesting a possible neoplastic transformation of FDCs within Castleman lesions [1, 4].

FDCS accounts for approximately 0.4%–0.8% of all soft-tissue sarcomas and typically affects adults in the third to fifth decades of life, with no marked sex predilection. The tumor most commonly arises in cervical lymph nodes, followed by extranodal sites such as the tonsil, gastrointestinal tract, liver, and spleen [5].

CD is a heterogeneous group of lymphoproliferative disorders characterized by shared morphological features on lymph node biopsy [6]. Based on clinical presentation and disease course, CD is classified into unicentric CD (UCD), which involves a single lymph node, and multicentric CD (MCD), which is marked by lymphadenopathy in multiple nodes. While UCD is typically reversible [7–9], MCD is a systemic, progressive condition that is often fatal [9].

Although some reports suggest that FDCS can develop from CD, most cases arise sporadically. FDCS typically presents as a painless, slow-growing mass without associated symptoms [3].

## 2. Case Presentation

A 30-year-old patient was admitted to our perinatology clinic at 28 weeks of gestation during her first pregnancy. She was diagnosed with early-onset preeclampsia, presenting with hypertension and proteinuria.

The patient had no history of chronic disease, medication use, or prior surgery.

Following hospitalization, obstetric ultrasonography revealed fetal growth restriction, with the fetal abdominal circumference at the 3rd percentile and an estimated fetal weight of 775 g. In addition, Doppler assessment showed absent end-diastolic flow in the umbilical artery and diastolic notches in both uterine arteries.

**Figure 1:**
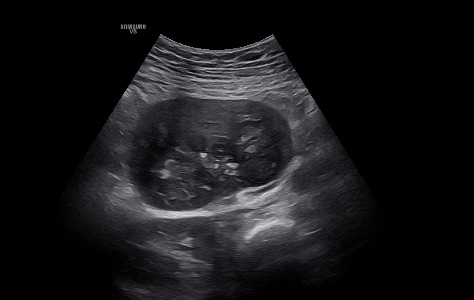
Preoperative image of the intraabdominal mass.

During ultrasonographic examination, an 80 × 70 mm solid mass with irregular borders and central calcification was incidentally detected in the right adnexal region. Based on these imaging characteristics, the lesion was initially interpreted as a solid mesenchymal tumor. The preliminary differential diagnoses included an ovarian nonepithelial tumor, a gastrointestinal stromal tumor (GIST), or a metastatic lesion. Given its relatively well-defined borders and lack of overtly malignant radiologic features, a low-grade neoplasm was considered more likely before histopathological confirmation (Figure 1). The patient was informed about the finding and advised of the possibility of oophorectomy during cesarean delivery if the mass originated from the ovary.

Following the diagnosis of the abdominopelvic mass, MRI imaging was planned. However, due to spontaneous decelerations and absent variability on the nonstress test, an emergency cesarean section was performed due to fetal distress.

The abdomen was accessed via a Pfannenstiel incision, and cytological sampling was performed by instilling 200 cc of saline into the abdominal cavity. The uterus was then entered through a Kerr incision, and a live male infant weighing 835 g and measuring 35 cm was delivered, with Apgar scores of 6 and 8 at 1 and 5 min, respectively. The uterus was closed in a single layer, and bleeding control was performed. Both ovaries and fallopian tubes appeared normal.

**Figure 2:**
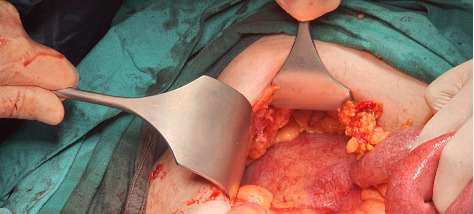
Intraoperative image of the intraabdominal mass.

A semimobile, solid mass measuring approximately 10 cm was identified in the retroperitoneum, adjacent to the lower part of the liver, extending across the midline from right to left (Figure 2). A general surgeon was consulted for further evaluation. Following the completion of preoperative radiological and laboratory assessments, treatment planning was deemed appropriate.

Postoperative contrast–enhanced abdominal computed tomography revealed a heterogeneous solid mass measuring approximately 69 × 98 mm within the mesenteric fat tissue on the right side of the midline. Multiple lymphadenopathies, the largest measuring 35 mm in diameter, were observed in the para-aortic and interaortocaval regions, as well as within the mesenteric fat tissue.

Cytological analysis showed no evidence of malignant cells.

**Figure 3:**
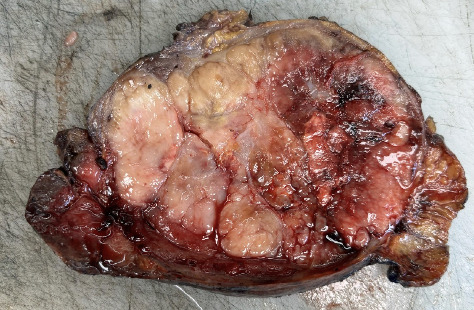
Gross specimen of the excised mass measuring 13 × 8 × 7.5 cm, dirty-white in color. The cut surface is dirty-white, multilobulated, and shows focal necrotic and hemorrhagic areas.

**Figure 4:**
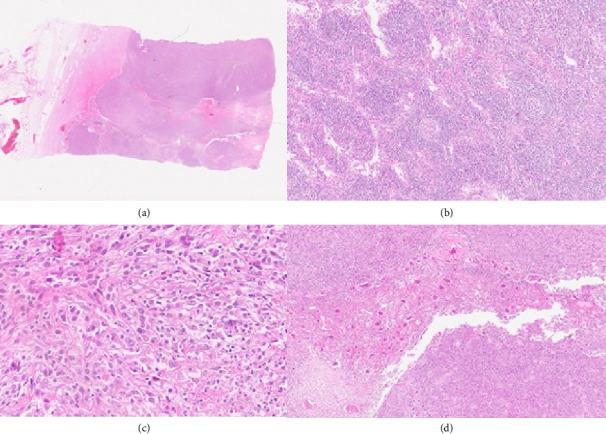
Histopathological findings of the resected mesenteric mass. (a) Low-power photomicrograph showing a well-circumscribed, solid, and lobulated tumor separated from surrounding mesenteric adipose tissue (H&E, × 40). (b) Lymphoid tissue exhibiting hyaline-vascular type Castleman disease, with germinal centers penetrated by hyalinized blood vessels, the classic “lollipop” sign (H&E, × 200). (c) Follicular dendritic cell sarcoma, composed of spindle-to-epithelioid neoplastic cells with irregular nuclear contours, vesicular chromatin, and prominent nucleoli arranged in fascicular and whorled patterns (H&E, × 400). (d) Atypical multinucleated giant cells scattered among spindle tumor cells (H&E, × 100).

On the 27th postpartum day, the patient underwent surgery by the general surgery team. Macroscopically, the excised mass measured 13 × 8 × 7.5 cm and appeared dirty-white in color, multilobulated, and partially hemorrhagic with focal necrotic areas. On sectioning, the cut surface was solid, lobulated, and pale to dirty-white, separated from the surrounding mesenteric adipose tissue (Figure 3). Microscopically, the tumor was well-circumscribed, solid, and lobulated, separated from the surrounding mesenteric adipose tissue (Figure 4(a)). In areas of lymphoid tissue, hyaline-vascular type CD was identified, characterized by germinal centers penetrated by hyalinized blood vessels forming the classic “lollipop” appearance (Figure 4(b)).

The neoplastic component consisted of spindle-to-epithelioid cells arranged in fascicular and whorled patterns with irregular nuclear contours, vesicular chromatin, and prominent nucleoli (Figure 4(c)). Atypical multinucleated giant cells were also observed (Figure 4(d)).

The background showed a dense infiltrate of lymphocytes and plasma cells, sometimes forming nodular aggregates. Mitotic activity ranged from 8 to 19 per 10 high-power fields, including atypical figures, and focal necrosis was present.

Immunohistochemistry demonstrated diffuse membranous and focal cytoplasmic positivity for CD21, D2-40 (podoplanin), and clusterin, confirming follicular dendritic cell lineage. CD34 was negative in tumor cells, though a rich vascular network with slit-like vessels was highlighted in the background. Ki-67 labeling index was approximately 30%–35%. Other markers including EMA, CD20, CD2, CD45, CD163, pancytokeratin, S100, SMA, desmin, STAT6, CAMTA1, progesterone receptor, estrogen receptor, CD117, and DOG-1 were negative. EBER in-situ hybridization for EBV was also negative.

Histopathological examination confirmed the diagnosis of FDCS arising in association with hyaline-vascular-type CD. The authors thank Prof. Dr. Güldal Esendağlı and her team, Department of Pathology, Faculty of Medicine, Gazi University, Ankara, Turkey, for their valuable contribution to the histopathological evaluation of this case.

## 3. Discussion

FDCS is a rare tumor, with only one previously reported case in a pregnant woman in the literature. In that case, a 20-year-old pregnant patient was diagnosed with FDCS after presenting with a painful mass in the right axilla. No obstetric complications occurred during pregnancy follow-up. Labor was induced at 34 weeks of gestation due to metastatic involvement of the mother's T12-L1 vertebrae and the need for chemotherapy [10].

To date, no reports in the literature have described an association between FDCS and obstetric complications such as preeclampsia and fetal growth restriction, as observed in our case.

Regardless of the clinical presentation, the uterus, adnexal structures, and cervix should be evaluated during standard diagnostic obstetric ultrasound examinations [11]. However, detecting abdominopelvic masses in pregnant women is challenging due to uterine enlargement. In our case, the mass was identified during an obstetric ultrasound at 28 weeks of gestation, and through multidisciplinary collaboration, appropriate treatment was provided.

The key takeaway from this case report is that obstetricians should remain vigilant for potential pelvic and abdominal masses when evaluating pregnant patients.

## Data Availability

The data supporting the findings of this study are available from the corresponding author upon reasonable request. These data are not publicly accessible due to confidentiality concerns and the potential risk of compromising the privacy of research participants.
